# Risk and benefit of protocol sedation and daily interruption of sedation for mechanically ventilated patients: a systematic review

**DOI:** 10.1186/cc12320

**Published:** 2013-03-19

**Authors:** K Hosokawa, M Egi, M Nishimura

**Affiliations:** 1 Kyoto Prefectural Yosanoumi Hospital, Kyoto, Japan; 2 Okayama University Hospital, Okayama, Japan; 3 Tokushima University Hospital, Tokushima, Japan

## Introduction

Protocol-directed sedation (PS) and daily interruption of sedation (DIS) are two major strategies to optimize sedation depth for mechanically ventilated patients. To clarify the clinical risk and benefits of PS and DIS, we performed a systematic review of randomized controlled trials (RCTs) in previous literature.

## Methods

We searched the MEDLINE database from January 1990 to October 2012 for studies on PS and DIS in critically ill patients. English-language manuscripts were included when they assessed the impact of PS or DIS on outcomes in critically ill patients requiring mechanical ventilation for >48 hours. PS is defined as sedation practices implemented by ICU staff using a sedation scale and a written protocol. DIS is defined as strategies with cessation of the continuous sedatives.

## Results

We retrieved 902 citations. Among them, nine RCTs fulfilled inclusion criteria. PS was compared with the standard practice in one RCT, DIS with the standard practice in four, PS with DIS in two, and PS with PS plus DIS (PS/DIS) in two. PS or DIS reduced the duration of mechanical ventilation by 28.9% and ICU stay by 27.8% compared with the standard practice. Neither the duration of mechanical ventilation nor ICU stay differed among DIS, PS and PS/DIS. Mortality did not differ among the three strategies. DIS increased the daily dose of sedatives and the workload of nurses in one recent RCT.

## Conclusion

PS or DIS decreased the duration of mechanical ventilation and ICU stay. PS seems to be superior to DIS based on the doses of sedatives and the workload of nurses.

